# Lemon grass essential oil improves *Gladiolus grandiflorus* postharvest life by modulating water relations, microbial growth, biochemical activity, and gene expression

**DOI:** 10.1038/s41598-023-28829-0

**Published:** 2023-02-14

**Authors:** Meenakshi Thakur, Vipasha Verma, Anjali Chandel, Raghawendra Kumar, Tanvi Sharma, Akhil Kumar, Sonali Bhardwaj, Rakshak Kumar, Bhavya Bhargava

**Affiliations:** 1 Floriculture Laboratory, Agrotechnology Division, CSIR-Institute of Himalayan Bioresource Technology, Palampur, India; 2 Academy of Scientific and Innovative Research (AcSIR), Ghaziabad, Uttar Pradesh 201002 India; 3 Biotechnology Division, CSIR-Institute of Himalayan Bioresource Technology, Palampur, India

**Keywords:** Biological techniques, Physiology, Plant sciences

## Abstract

Gladiolus (*Gladiolus grandiflorus* Andrews) is a high-valued bulbous cut flower. However, the shorter postharvest life of the gladiolus, limits its marketing and commercial value. In the present investigation, the effect of lemon grass (LG) essential oil as an antimicrobial agent was studied towards increasing the vase life of gladiolus. The results revealed that as compared to control (distilled water), treatment with a lower concentration of 5 µL L^−1^ LG essential oil prolonged the vase life of gladiolus up to 11 days (d). Scanning Electron Microscope (SEM) observation indicated that the sample treated with 5 µL L^−1^ LG essential oil showed intact vasculature, suggesting reduced microbial blockage at the stem end which was further corroborated by microbial count. Biochemical analysis suggested an increased level of total soluble sugars, carotenoid content, lower MDA accumulation, and higher activity of antioxidant enzymes in LG treated flowers. Moreover, transcripts levels of genes associated with senescence viz.*, GgCyP1* and *GgERS1a* were downregulated, while expression of *GDAD1* and antioxidant genes such as *GgP5C5, GgPOD 1, GgMnSOD,* and *GgCAT1* were upregulated in LG treated cut spikes as compared to control. Among various treatments we have concluded that, the vase life of the gladiolus cut spike was improved along with the relative fresh flower weight and diameter of flower at the lower dose of 5 µL L^−1^ LG oil in the vase solution. Thus, LG oil as an eco-friendly agent has the potential to extend the postharvest life of cut flowers.

## Introduction

Gladiolus (*Gladiolus grandiflorus* Andrews), a member of the Iridaceae family, is an important bulbous cut flower that originates in Cape Provinces in South Africa. It is one of the high valued bulbous cut flower crop in the global flower market. The wholesale value of gladiolus was $20.175 M in the United States^3^ whereas in China the value was approximately $41M^1^. In India the total cultivation area of gladiolus is about 11,660 ha with an estimated production of 1060 million cut flowers. However, the total export of gladiolus from India was 10,165.28 MT with value of approximately 138 million INR^2^. The availability of gladiolus flowers in various colors makes them popular for flower arrangement, bouquets, and bedding of gardens. However, to keep a continuous supply in the market longer shelf life of gladiolus florets is desired by the consumers. Therefore, one of the main challenging tasks in floriculture for postharvest researchers is to extend the shelf life of cut flowers like gladiolus for distant marketing. Pre-harvest life of cut spikes is dependent upon various factors like environment, genetics, management, and harvest time. However, post-harvest life is determined by maintained water relations, microorganisms influence, storage conditions, and packaging practices. Microbes have the ability to rapidly grow and colonize at the stem end of postharvest cuttings which consequently lead to the blockage of xylem vessels. As a result of microbial blockage, the transport and water uptake get disrupted, ultimately causes water imbalance and early wilting in cut flowers^3^. Thus, to avoid early senescence in cut flowers, prevention of microbial blockage is an useful approach to increase the post-harvest quality of flowers. The shelf life of cut flowers has been improved by the inclusion of different chemicals such as silver nitrate, silver thiosulfate, nano silver, calcium and hydrogen gas^4–6^. However, the high cost of these chemicals and realizing their negative impact on environment and human health have diverted researcher’s attention towards the use of eco-friendly agents. As vase life is determined by water relationships, the pace of senescence can be slowed down by applying antibacterial preservative solutions.

Essential oils play an important role in postharvest management of crops and its produce to protect plants due to their antifungal, antiviral, antibacterial, and insecticidal properties. Lemon grass (LG), also known as *Cymbopogon citratus* (D.C) stapf, is a member of the Poaceae family^7^. The main ingredients of LG essential oil include neral, isoneral, geranial, isogeranial, geraniol, geranyl acetate, citronellal, citronellol, germacrene-D, and elemol. Among them, bioactive constituents like α-citral (geranial) and β-citral (neral) has demonstrated their antimicrobial activity by suppressing the growth of several bacteria^8^. Previous studies have reported the positive effect of different plant extracts, and essential oils in increasing the vase life of cut flowers viz., gerbera, gladiolus, alstroemeria and roses^9–12^. The process of petal senescence is highly complex and includes a series of physiological and biochemical changes such as an alteration in cell membrane permeability, which leads to pigment destruction, wilting and eventually petal senescence^13^. Many senescence-associated genes encoding cysteine proteases and lipoxygenases get upregulated during petal senescence^14^. In various ethylene-sensitive cut flowers, ethylene is the key regulator of petal or floral senescence. Endogenously produced ethylene triggers senescence and coordinates gene expression in flower petals^15^. Gladiolus, on the other hand, is an ethylene-insensitive flower, and petal senescence in such cut flowers can be caused by endogenous cues other than ethylene such as the increase in reactive oxygen species (ROS). Antioxidant enzymes like peroxidase, catalase, superoxide dismutase (SOD), and ascorbate oxidase act as scavengers of reactive oxygen radicals and imparts defense^16^. The purpose of this study was to define the role of LG essential oil as a preservative treatment to reduce the senescence of gladiolus cut flowers. As the effect of any chemical or biological agent is concentration-dependent, it becomes important to determine the effective concentration of LG essential oil that produces the quality cut flower with longer vase life. Therefore, the present study has been undertaken to determine the optimal doses of LG essential oil for the enhancement of vase life and quality of gladiolus by regulating water relations and keeping the stem vasculature intact. Also, the results were substantiated by the microbial count, biochemical activity, and senescence-associated gene expression.

## Materials and methods

### Planting material

The corms of *Gladiolus grandiflorus* cv. Nikita were collected in the month of June, 2020 from the established nursery at Council of Scientific and Industrial Research (CSIR)—Institute of Himalayan Bioresource Technology (IHBT), Palampur, Himachal Pradesh India for the experimental purpose. After collection, these corms were stored at 7 °C and cultivated and maintained in the month of February, 2021 at the experimental farm of Agrotechnology Division at CSIR-IHBT, Palampur, Himachal Pradesh India [Latitude (32 6′ 52 N); Longitude (76 33′ 24E); altitude of 5298 ft; mean annual rainfall 2493 mm; average annual temperature 19.1 °C]. All the protocols including experimental research and collection of plant material were complied with relevant institutional, national, and international guidelines and legislation. Morphological uniform and healthy flower spikes at the stage of first colored bud appearance were chosen for the current study. The cut spikes of gladiolus were harvested in the morning 8:00–9:00 A.M. during July 2021. After harvesting, these cut spikes were immediately placed in a bucket of distilled water and transferred to the laboratory. Each spike was recut under distilled water up to 60 cm in length before the experiment execution. Spikes bearing 10–12 flower buds were selected for the study and from each spike leaves were removed. On the first day of experiment, each spike was placed in the individual vase containing 500 mL of vase solution, and not renewed further during the vase period.

### Experimental details

The vase life assessment experiment was carried out at room temperature 28.5 ± 1.6 °C, relative humidity (RH) of 72.9 ± 2.3% under a daily photoperiod of 12 hour (h) with the illumination of 10–15 μmol m^−2^ s^−1^. LG essential oil was taken from the Floriculture Lab of Agrotechnology Division of CSIR-IHBT, Palampur, Himachal Pradesh. The current study examined the influence of LG essential oil at concentrations of 0, 5, 10, 20, 30, 40, 50, and 75 µL L^−1^ on the vase life of gladiolus. The essential oil and emulsifier (dimethyl sulfoxide) were mixed at a volume ratio of 1:4 to create a pre-concentration of LG essential oil in emulsion form. Afterwards, the stock emulsion was diluted with distilled water to make treatment solutions ranging from 0 to 75 µL L^−1^ LG essential oil. Postharvest performance was compared with mock containing distilled water and 0.03% DMSO solution, referred to as control. In total, 21 cut spikes from each treatment in three replications (seven spikes for each replication) were taken for the experimental study.

### Relative water uptake, pH, floret opening, floret diameter, relative fresh weight, and vase life assessment

Water uptake was expressed as relative water uptake (mL) calculated as difference between the volume of vase solution on the initial day and on measurement day. The pH of different vase solutions was measured by using a pH meter (model Eutech Instruments pH 510) and the floret opening rate was determined by using the formula: Percentage of opened florets/total number of florets (tight bud to fully opened flower stage). A digital caliper gauge was used to measure the diameter of the first, third, and fifth floret of a cut spike. Change in fresh weight (%) was represented as the relative fresh weight of the whole cut spike. For physiological analyses, all the measurements were done at 5, 7, and 11 days (d) of the evaluation period. Vase life of cut spikes was calculated from the time of harvest to the time when half of the total florets get wilted due to loss of their turgor and as on the onset of petal senescence (≥ 50% petal drop)^23^.

### Bacterial isolation

The bacterial isolation was performed by using the method described by Balestra et al.^17^ and Li et al.^18^, with some modifications. Briefly, the end part of the gladiolus spike was cut with the help of sterile scalpel blades and made into small pieces. These pieces were placed in sterile tubes with 1 mL of sterile 0.9% normal saline. The small pieces were gently crushed by using the sterile motor pestle to dislodge the bacteria. Up to 10^–10^ serial dilutions of 100 µL aliquot of the liquid extract was prepared with sterile 0.9% normal saline. From each dilution, 0.1 mL was taken and spread onto nutrient agar plate, and then incubated for 24 h at 37 °C. After 24 h of growth, bacterial colonies from the plates were picked and streaked on the same media containing plate until pure colonies were obtained. Pure bacterial isolates were preserved in – 80 °C freezer (Eppendorf, INDIA) in 25% glycerol for further use.

### DNA isolation and molecular identification

The fresh single bacterial colony was inoculated in 10 mL nutrient broth and incubated overnight in an orbital shaker (180 rpm) at 37 °C. Centrifugation at 11,200*g* for 5 min was used to collect the cells. A commercial kit called the Pure Link Microbiome DNA purification kit (Thermofisher, USA) was utilized to extract genomic DNA. The integrity of genomic DNA was tested on a 0.8% agarose gel, and the purity and concentration of DNA were evaluated using a Nanodrop 1000 Spectrophotometer (ND-1000 Thermo Scientific, USA). After that, the amplification of the 16S rRNA gene from isolated DNA was done using bacterial universal primer set 27F (5′-AGA GTT TGA TCM TGG CTC AG-3′) and 1492R (5′-TAC GGY TAC CTT GTT ACG ACT-3′). The 25 µL PCR master mix consisted of 2.5 µL of 10× PCR buffer, 0.5 µL 10 µM of each primer, 2.5 µL dNTPs, 0.25 µL Taq polymerase (Sigma-Aldrich, USA), 1–2 µL DNA template and the total volume was made upto 25 µL using ddH_2_O. The PCR amplification was performed in ProFlex PCR System (Applied Biosystems, United States) thermal cycler using optimized conditions^4,19^. The PCR amplified products were analyzed on 1.0% agarose gel and purified using the ExoSAP (Applied Biosystems, United States) and sent for sequencing using both forward and reverse primer. The full-length sequence was aligned with gene sequences available in (NCBI + DDBJ + EMBL) using the Bio-Edit software, which aligned both the forward and reverse sequences. The putative phylogenetic affiliation was determined with 95% confidence using the naive Bayesian rRNA classifier and the RDP-II database^20^. The phylogenetic tree was created in MEGA X using Neighbor-joining with a 1000 bootstrap value^19^.

### Scanning electron microscopy (SEM) observations

On the 5 and 7 day of the vase period, stem segments (1 cm in length) were excised from the base of each spike (control and LG treated stems) using fresh surgical blades. The stem segment samples were promptly fixed in formalin-acetic acid-alcohol (FAA) and stored overnight. The samples were then dehydrated for 10 min in a series of ethanol concentrations (25, 50, 70, 85, and 100%). The dehydrated samples were then dried and coated with gold–palladium. Finally samples were analysed under Scanning electron microscope (SEM) (JEOL, Ltd, Tokyo, Japan) to observe bacterial proliferation in xylem vessels^21^.

### Biochemical assays

Petal samples of the fourth floret from the lower side of the spike were taken on 0, 5, 7, and 11 days of the vase period and were stored at − 80 °C for biochemical and enzyme assays.

### Analysis of total soluble sugars and pigments

Total soluble sugars in flower petals during vase life were determined by using the method cited by Gilmour et al.^22^. Flower petals (100 mg) were grounded to a fine powder in liquid nitrogen. To the grounded powder, 2 mL of 80% (v/v) ethanol was added and subsequently, the samples were incubated at 80 °C for 15 min. The samples were shaken for 1 h at room temperature then they were stored at 4 °C overnight. Debris was separated by centrifugation for 5 min at 11,200*g*. Samples were shaken with water and chloroform in 1:1 ratio for 10 min. The aqueous phase was assayed for total soluble sugar content using the microplate-based phenol–sulphuric acid as per the method described by Masuko et al.^23^. A microplate reader was used to measure the absorbance of the samples at 490 nm (Synergy H1 BioTek, USA) and the results were expressed as mg g^−1^ of fresh weight. The carotenoid content of the petal was measured using Kirk and Allen's^24^ method of extraction in 80% acetone. The absorbance of extracts was measured at 480 nm, 663 nm and 645 nm respectively, using a microplate reader (Synergy H1 BioTek, USA), and the results were expressed as mg g^−1^ of fresh weight (FW).

### Malondialdehyde (MDA) content

Lipid peroxidation in flower petals during the vase period was determined by quantifying malondialdehyde (MDA) content^25^. Frozen petal tissues (200 mg) were grounded in liquid nitrogen to a fine powder and mixed with 0.5% (w/v) thio-barbituric acid, 20% (v/v) tri-chloroacetic acid, 0.25 mL of 175 mM NaCl in 2 mL of 50 mM Tris–Cl, pH 8. The reaction solution was incubated at 100 °C for 10 min followed by cooling. A microplate reader was used to measure the absorbance of the supernatant at 450, 532, and 600 nm (Synergy H1 BioTek, USA). MDA content was expressed as μmol g^−1^ of fresh weight.

### Superoxide dismutase (SOD) and catalase (CAT) activity assay

Activities of antioxidant enzymes were measured during the vase period. Petal samples (200 mg) were stored at − 80 °C and were grounded in 2 mL of 50 mM phosphate buffer pH 7.8 at 4 °C followed by centrifugation at 13,000*g* for 10 min. The supernatant was used to measure SOD (EC 1.15.1.1) and CAT activities (EC 1.11.1.6). The activity of SOD was assayed according to the method by Beyer and Fridovich^26^. The reaction solution for SOD contained 1.17 μM riboflavin, 0.57 μM *p*-nitro blue tetrazolium chloride (NBT), 0.025% Triton X-100, and 9.9 mM methionine in 50 mM potassium phosphate buffer (pH 7.8) and enzyme extract^27^. The reaction solution was exposed to light (150 μmol^−1^m^2^ s^−1^) for 10 min at 25 °C and thereafter absorbance was recorded using a microplate reader at 560 nm. The activity of CAT was measured following the method of Klapheck et al.^28^. The reaction solution for catalase (CAT) was composed of 15 mM H_2_O_2_, 50 mM phosphate buffer, and enzyme extract. The decrease in absorbance of H_2_O_2_ was recorded for 1 min at 240 nm. The results were expressed as unit mg^−1^ protein.

### Gene expression

On the 5 d of post-treatment, 100 mg of petal tissue was collected and the total RNA was extracted using the IRIS method^29^. Thereafter, using 1 μg of total RNA, the cDNA was synthesized using an oligo dT20 primer and a reverse transcription kit (Verso cDNA Synthesis Kit, Thermofisher Scientific, USA). For real-time PCR, the constructed cDNA was used as a template with specific primers for senescence-associated genes and genes encoding antioxidant enzymes. The quantification of gene expression was carried out in the ABI Real-Time machine (Applied Biosystems step-one Thermofuse USA). Each reaction was carried out in three replicates. The transcript levels of the petal senescence-associated genes and antioxidant enzymes encoding genes were calculated using a double standard curve with the following thermal profile program which involved, the initial stage of 10 min at 95 °C; 40 cycles of 15 s at 95 °C, and 1 min at 60 °C. After amplification, a melting curve (65–95 °C with an increment of 0.3 °C) was generated for each reaction to verify the specific amplification. The expression levels of target mRNAs were normalized to Actin (internal control gene) and were calculated using the 2^−ΔΔCt^ method^30^. The primers used for quantifying the expression levels of genes were given in Table 1.

**Table 1:** Gene code numbers and primer sequences used for transcript quantification by RT-PCR.

*GgCyP1 F*	ATGCTCCTACTAGCCCTAGTCTTTCTTGC
*GgCyP1 R*	ACGTAATGCCTCAAATCCACCTCTCTTCC
*GgERS1a F*	ATGGAGGGATGTGATTGCATCGAGCCGCA
*GgERS1a R*	CTCCCGACGAGCTAAGTCTAGGGCAACA
*GgDAD1 F*	ATGGCAAAATCAACTGCTAAT
*GgDAD1 R*	TTATCCAAGGAAGTTCATGAT
*GgP5CS F*	ATGGCAGTTTCAGCAAGGGA
*GgP5CS R*	ATCCACTTCTGGTTCGCCTC
*GgMnSOD F*	AACTACAACACCGCCCTAGC
*GgMnSOD R*	CCCTCACTGACAGGCTTGAG
*GgPOD1 F *	CCAGCTCGCTCACGATAAGT
*GPOD1 R*	ATCTCCCCCTTGGTTCCAGT
*GgCAT1 F*	GATGGGTGGATGCTCTGTCA
*GgCAT1 R*	CCAGTTTCTGGCCCAACGAT
*GgACT F*	ACTGCAGAGCGGGAAATTGT
*GgACT R*	CCAATCAGGGATGGCTGGAA

### Statistical analysis

Data were provided as means with standard errors (SE) of three replicates per treatment. Using SPSS version 19 (IBM), the data were subjected to analysis of variance (ANOVA) at *P* = *0.05*. Physiological, biochemical, and gene expression data were represented at different concentrations of LG oil (0, 5, 30, and 75 µL L^−1^). The mean values were compared by the Tukey's test at the 5% level of probability.

## Results

### Water uptake and pH

**Figure 1:**
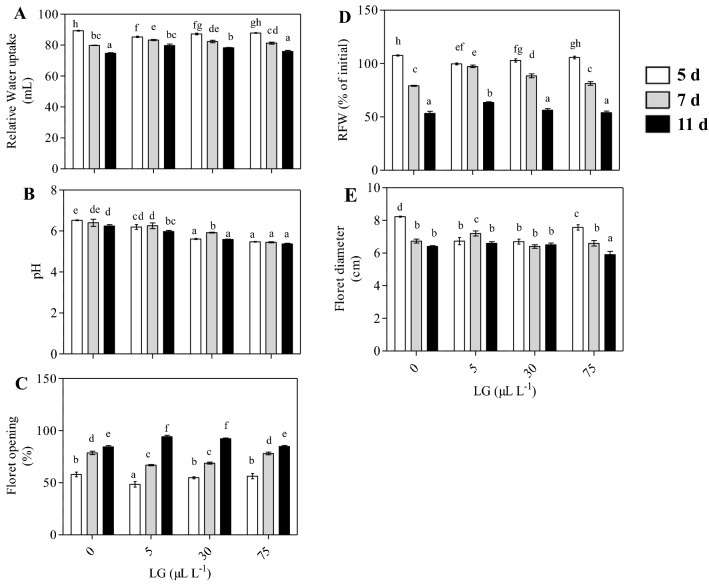
Morpho-physiological parameters such as relative water uptake (**A**), pH (**B**), floret opening (**C**), floret diameter (**D**), relative fresh weight (**E**), in untreated control and LG essential oil treated *Gladiolus grandiflorus* spikes at different vase days (d) of evaluation. The gladiolus spikes were treated with different LG essential oil concentration (0, 5, 30 and 75 µL L^−1^). Each value is the mean ± SE of three replicates. Means with different letters are significantly different at 5% probability (Tukey’s test). *Floret Diameter: Average of first, third and fifth floret was taken.

Relative water uptake in both treated and untreated cut spikes decreased progressively with an increase in postharvest days (Fig. 1A). At 5 days of evaluation, the lowest water uptake was observed in cut spikes treated with 5 µL L^−1^ LG essential oil solution. However, during the later days of vase life, LG essential oil (5µL L^−1^) treated spikes followed a reversible trend by alleviating this decline and maintaining the highest water uptake as compared to control and other concentrations (Fig. 1A). The pH of the solution decreased significantly with an increase in concentrations of LG essential oil. Maximum pH was reported in the vase solution without LG essential oil. The highest pH was observed on the fifth day thereafter it showed decreasing trend till eleventh day of observation (Fig. 1B).

### Floret opening (%) and floret diameter (cm)

The increasing trend in floret opening was observed with the increase in the number of vase days. However, variation in floret opening rate was observed within the treated and untreated cut spikes. Upon evaluation of different vase days, the highest floret opening rate was observed in untreated cut spikes. Early floret opening in control was directly correlated with physiological process of senescence. Whereas, the lowest floret opening rate was recorded in 5 µL L^−1^ LG solution (83.5%) even at 11 days of evaluation, led to delayed opening of flowers thus determining the role of LG essential oil in increased vase life of gladiolus (Fig. 1C). To evaluate change in floret diameter among treated and untreated cut spikes average of the first, third and fifth floret was taken. Initially, at 5 days of evaluation, the increment in flower diameter was observed in the untreated cut spike as compared to LG treated cut spikes (Fig. 1D). However, on later vase days, LG treated cut spikes (5 µL L^−1^) showed increased floret diameter of pre-selected florets (Fig. 1D).

### Relative fresh weight (RFW%) and vase life (days)

**Figure 2:**
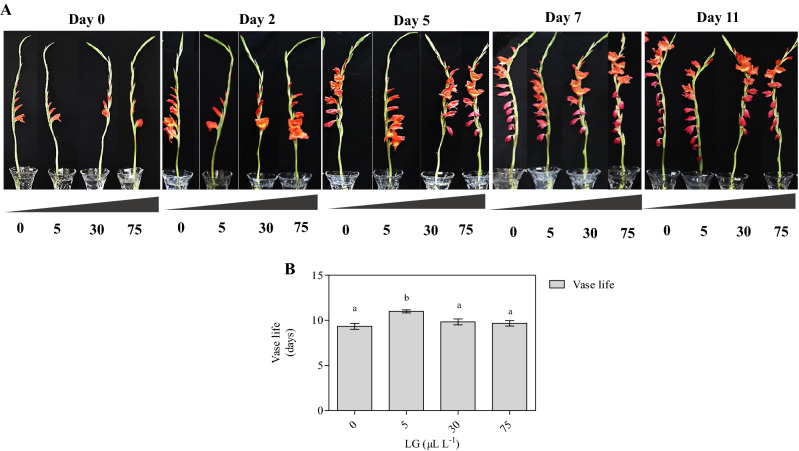
Representative photographs of vase performance (**A**), vase life (**B**) of gladiolus in untreated (control) and LG essential oil treated spikes. The *Gladiolus grandiflorus* spikes were treated with different LG oil concentration (0, 5, 30 and 75 µL L^−1^). Each value is the mean ± SE of three replicates. Means with different letters are significantly different at 5% probability (Tukey’s Test).

Generally, RFW decreased significantly with an increase in vase days. Upon 7 days of evaluation, RFW within the treatments was significantly higher in cut spikes placed in 5 µL L^−1^ (97.3%) LG essential oil solution as compared to control (79.2%). A similar trend was observed at 11 days of observation (Fig. 1E). Compared to control, the effects of different concentrations of LG essential oil on the vase life of cut gladiolus were significant and dose-dependent (Fig. 2B). The vase life of gladiolus was extended by a lower dose of LG essential oil at 5 µL L^−1^ solution, whereas control and higher concentration of LG essential oil showed reduced vase life and early senescence with more loss of visual quality. The maximum vase life of 11 days was recorded in cut spikes placed in 5 µL L^−1^ LG essential oil compared to control (9 days) (Fig. 2).

### Bacterial count and identification

**Figure 3:**
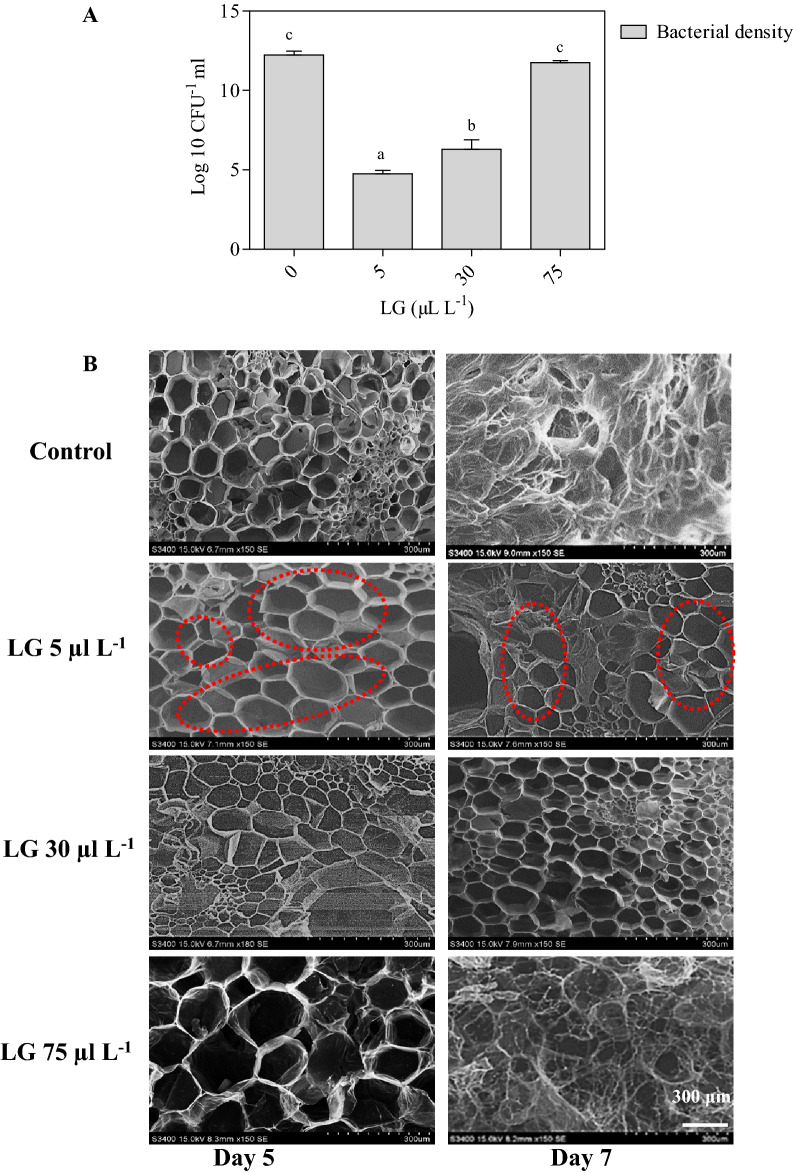
(**A**) Bacterial count in untreated (control) and LG essential oil (5, 30 and 75 µl L^−1^) treated cut spikes of *Gladiolus* at 5th day. Each value is the mean ± SE of three replicates. Means with different letters are significantly different at 5% probability (Tukey’s test). (**B**) SEM characterization of stem ends of untreated and LG essential oil (5, 30 and 75 µl L^−1^) treated spikes of *Gladiolus grandifloras* Observations were carried out on 5th and 7th day. Scale bars: 300 μm. Red circle indicates intact vasculature with lesser blockage.

The relationship between bacteria and vase life of gladiolus is revealed by the number of bacteria present in the stem segments. It was observed that the density of bacteria in the stem ends of control spikes was higher (1.08 log ^10^ CFU mL^−1^) compared to the LG treated samples. However, LG essential oil at 5 µL L^−1^ concentration in the vase solution has reduced the bacterial load (0.67 log ^10^ CFU mL^−1^) (Fig. 3A). Based on phenotype, we observed only one type of bacteria present in the cut spike of gladiolus*.* The SEM observation revealed that 5 µL L^−1^ LG solution has resulted in the reduced bacterial colonization and biofilm formation on the xylem vessels of cut gladiolus spikes consequently, retaining the intact vasculature in the stem of gladiolus (Fig. 3B).

### Phylogenetic analysis of 16S rRNA

**Figure 4:**
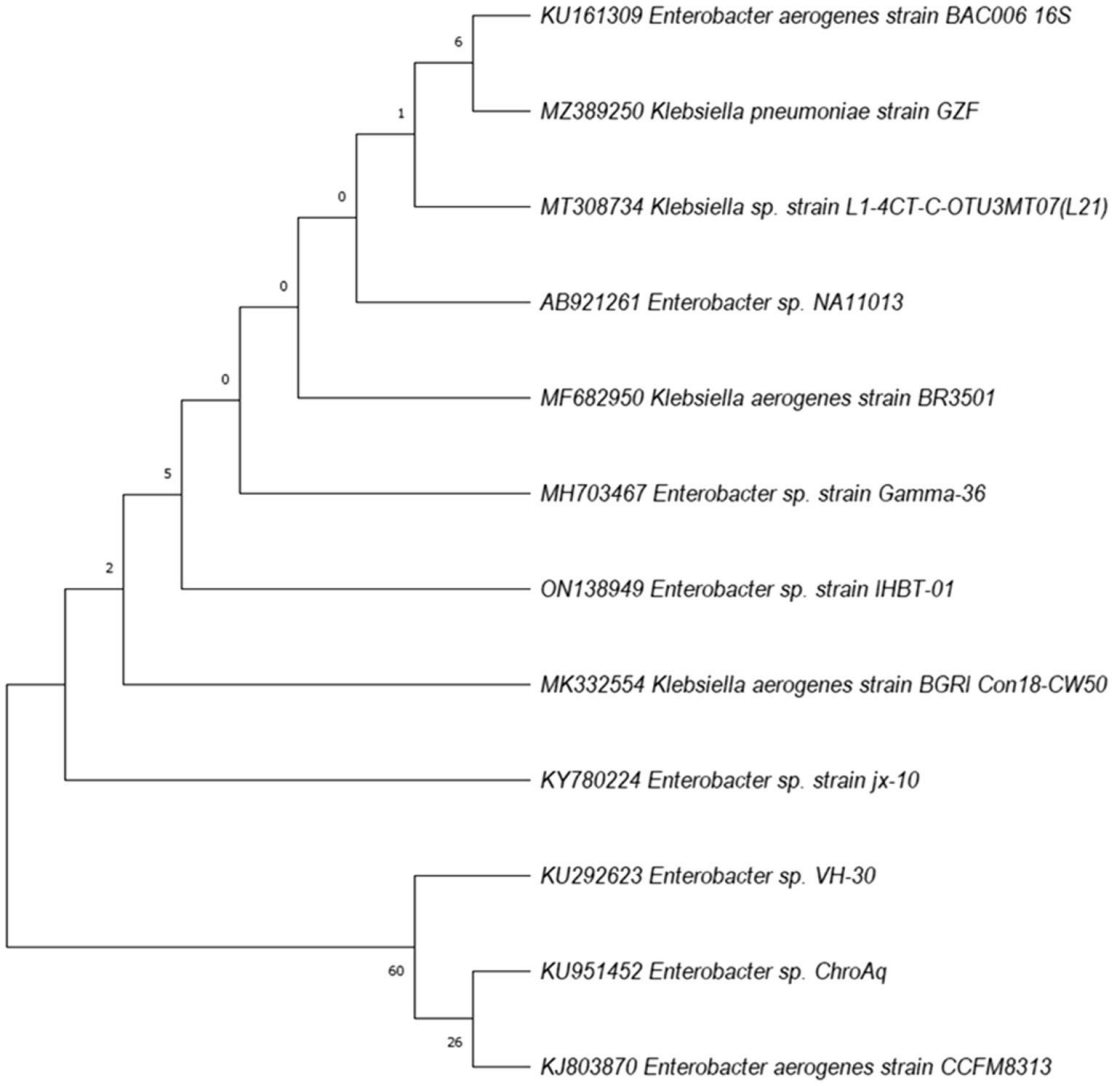
The evolutionary history was inferred by using the Maximum Likelihood method and Tamura-Nei model. The 16S rRNA gene sequences showing relationships between strain *Enterobacter* sp. and closely related members of the genus *Enterobacter*. Numbers at nodes indicate levels of bootstrap support based on a neighbor-joining analysis of 1000 resampled datasets; only values above 50% are given.

The sequence of the 16S rRNA gene was amplified and sequenced. Upon analysis, it was observed that the partial 16S rRNA gene sequence RD-covered 1356 bp having an average of 54.2% of GC content. Nucleotides were subjected to BLASTn analysis which showed 100.0% similarity and 99.8% identity with the *Enterobacter aerogenes* strain BAC006. A phylogenetic tree was created using the nucleotide sequences of the type strain which was retrieved from the NCBI. The phylogenetic position of the *E. aerogenes* strain IHBT-01 (ON138949) showed similarity with neared bacterial strain within a cluster that contains *Enterobacter* sp. strain jx-10 (KJ575043), *Enterobacter* sp. ChroAq. (KU951452), *Klebsiella aerogenes* strain BGRI Con18-CW50 (MK332554) and *Enterobacter* sp. VH-30 (KU292623) (Fig. 4). The 16s rRNA gene sequence has been submitted to gene bank (NCBI) under the accession No. ON138949.

### Biochemical assays

#### Total soluble sugars and carotenoids

**Figure 5:**
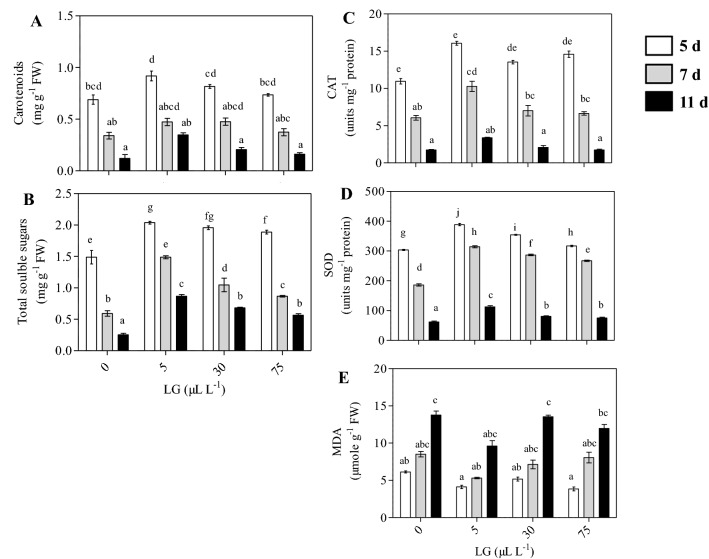
Total soluble sugar content (**A**), Carotenoid content (**B**), MDA (**C**), SOD activity (**D**), CAT activity (**E**), in the petals of untreated (distilled water) and LG essential oil treated gladiolus spikes. The Gladiolus spikes were treated with different LG essential oil concentration (0, 5, 30 and 75 µL L^−1^). Each value is the mean ± SE of three replicates. Means with different letters are significantly different at 5% probability (Tukey’s Test). *Petals from the fourth floret was used for the analysis.

Total soluble sugars in the flower petals gradually increased till the fifth day of the vase life period and thereafter it declined and was lowest on the last day of vase life. However, the highest concentration of total soluble sugars was observed on 11 days in flowers exposed to 5 µL L^−1^ LG essential oil during the vase period (Fig. 5A). The concentration of carotenoids decreased gradually in the petals of both treated and untreated cut spikes during the vase life (Fig. 5B). The extent of this decline was maximum in control flowers. Among all treatments, the decrease in carotenoid content was less pronounced in flowers exposed to 5 µL L^−1^ LG essential oil. This treatment also retained the highest carotenoid levels even on the eleventh day of vase life.

#### Malondialdehyde (MDA) content and antioxidant enzymes

The oxidative damage to the petals was assessed by measuring MDA content. MDA content gradually increased in flower petals during vase life. However, MDA content was lower in flowers exposed to different concentrations of LG essential oil as compared to control flowers at all the time points considered during vase life. Among all treatments, MDA content was lowest in flowers exposed to 5 µL L^−1^ LG essential oil during the vase period (Fig. 5C). The activities of antioxidant enzymes were influenced during the vase period. The activities first increased and thereafter declined and were lowest during the last day of vase life. Significant increase in the SOD (Fig. 5D) and CAT activities (Fig. 5E) were observed in the flowers exposed to different concentrations of LG essential oil in comparison to control. However, the highest activity of antioxidant enzymes was observed even on 11 days in flowers exposed to 5 µL L^−1^ LG essential oil during the vase period.

### Quantification of the genes related to petal senescence and antioxidant enzymes

**Figure 6:**
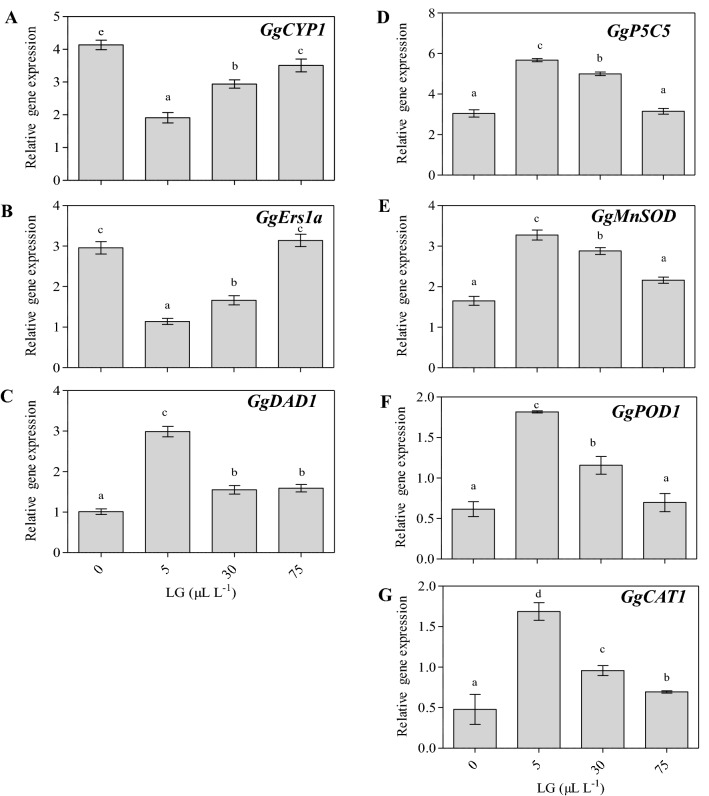
The transcripts levels of senescence associated genes *GgCYP1* (**A**)*, GgErs1a* (**B**), *GgDAD1* (**C**) and antioxidants encoding genes *GgP5C5* (**D**), *GgMnSOD* (**E**), *GgPOD1* (**F**) *GgCAT1* (**G**) in the petals of untreated (distilled water) and LG oil treated spikes ((5, 30 and 75 µL L^−1^) of *Gladiolus grandiflorus*. Each value is the mean ± SE of three replicates. Means with different letters are significantly different at 5% probability (Tukey’s Test).

For further validation of the effect of LG essential oil on vase life, the expression of different genes associated with senescence (SAGs) viz*., GgCyP1, GgErs1a,* and *GgDAD1* and genes encoding antioxidant enzymes such as *G*g*P5C5, GgPOD 1, GgMnSOD*, and *GgCAT1* was measured in untreated control and LG essential oil-treated spikes of gladiolus on 5 days of post-treatment. Among petal senescence-associated genes, the transcript levels of the genes *CYP 1* in the flowers were highest in the untreated control as compared to spikes treated with LG essential oil, whereas the lowest expression levels of both genes were recorded in the 5 µL L^−1^ concentration. (Fig. 6). LG essential oil has dramatically reduced the expression of *GgCyP1,* thereby retarding one of the crucial factors of senescence. The expression of *GgErs1a* also followed the same trend as *GgCyP1* and showed a reduction in transcript levels in the 5 µL L^−1^ LG essential oil-treated spikes in comparison to control. However, there was no such significant difference was found in the expression level of the *GgErs1a* between the untreated control and 75 µL L^−1^ LG treated spikes**.** Unlike *GgCyP1* and *GgErs1a* the expression of *GgDAD1* gene which refers to defender of apoptotic death was up-regulated by LG oil treatment in gladiolus but the maximum expression of *GgDAD1* was observed in 5 µL L^−1^ LG treated spikes as compared to control and other LG treated spikes (Fig. 6). Thus, our study suggests LG essential oil showed a role in the delay of senescence and may enhance vase life in gladiolus florets by downregulation *of GgCyP1, GgERS1a,* and up-regulation of *GgDAD1* genes.

The antioxidant gene expression analysis in the florets of LG treated spikes showed upregulation of *GgP5C5, GgPOD 1, GgMnSOD, and GgCAT1* as compared to untreated control spikes of gladiolus. However, the expression levels reached the maximum extent at 5 µl L^−1^ LG essential oil treatments in the case of *GgP5CS*, Gg*POD 1, GgMnSOD, and GgCAT1* genes. The gene expression levels of *GgCAT1* and *GgMnSOD* antioxidant enzyme-encoding genes in gladiolus were found to be consistent with the observed trend of the corresponding antioxidant enzyme activities of *GgCAT1* and *GgMnSOD* (Fig. 6)*.*

## Discussion

The use of plant secondary metabolites such as essential oil/plant extracts can play a pivotal role in promoting the shelf life of cut flowers. In our study, exogenous addition of LG essential oil has increased the postharvest longevity of cut spikes of gladiolus. Similar effects of other essential oils or natural extracts has also been reported in other cut flowers^16,31^. The most important factor that trigger postharvest senescence in cut flowers is water loss and reduced water uptake caused by bacterial xylem occlusion. Application of LG essential oil showed increase in vase life of gladiolus by maintaining proper water uptake, increased enzymatic antioxidants, and reduced oxidative damage due to its antimicrobial properties. In addition, LG essential oil at lower concentrations suppressed the expression of positive (*GgCYP1* and *GgERS1a*) and inversely upregulated the expression of negative (*GgDAD1*) regulators of senescence.

The increased vase life of gladiolus by the lower concentration of LG essential oil might be explained by well-maintained water balance (Fig. 1A). Previous studies have also reported improvement in water relations upon the application of natural extract in gladiolus^32,33^. Maintenance of regulated water uptake extensively increased the vase life of gladiolus, however, disturbance of water uptake leads to senescence. This means that gladiolus is sensitive to water shortages caused by disturbing the postharvest water balance^12^. The increased solution uptake might be attributed to the well-known antimicrobial activity of citral (aldehyde) present in the LG essential oil, which could keep the water free from bacteria and other microbes, and form an occlusion inside the stem that blocked water passage to the bloom. The mechanistic action behind the antimicrobial action of citral involve the destruction of bacterial biofilms which further hinders the bacterial growth and development^34^. Moreover, biofilm formation at the stem ends leads to the disruption of hydraulic conductivity resulting in reduced water uptake from the vasculature ultimately causing wilting/early senescence. LG essential oil components destabilizes the bond between the lipid bilayer and neutralize the bacteria through membrane disintegration^35^. The potential of LG essential oil specifically citral as promising agents against polymicrobial biofilms was further demonstrated by the transcriptional analyses which indicated citral mediated downregulation of genes involved in peptidoglycan, quorum sensing and fatty acids biosynthesis of *Staphylococcus aureus*^36^. Furthermore, other minor components such as limonene, linalool, and myrcene along with citral have a synergistic effect thus can play a major role in augmenting LG essential oil antimicrobial efficacy^37,38^. Therefore, reduced bacterial density in the cut spikes treated with exogenous application of a low dose of LG essential oil could be the additional factor for maintaining the water relation.

In addition, the pH of a preservative solution of LG essential oil showed a direct relationship with microbial growth. The current results revealed that freshly prepared solutions had comparatively similar pH in comparison to control, however, with the increase in vase days’ pH of the preservative solution decreased eventually (Fig. 1B). This might be due to the acidic nature of the microemulsions of LG essential oil^39^. The acidic condition of the microemulsion is reported to prevent microbial growth as microbes required higher pH for its growth and development^40^. Similar results have been reported in carnation, where the addition of essential oils to vase solution lowered pH and inhibited micro-organisms growth^41^.

During the storage and post-harvest life of cut flowers, delayed floret opening is often recommended. However, too much delay in floret opening might cause permanent bud opening failure, and too early opening can limit vase life due to early senescence and wilting. So, the opening of the floret at the appropriate time decides the vase life of spikes^42^. The inclusion of LG essential oil in vase solution slow down the process of floret opening but did not lead to its failure (Fig. 1C). The decreased floret opening in cut spikes can be due to the gradual antimicrobial action of LG essential oil and slower water uptake consequently. The trend of delayed floret opening and increased vase life have also been reported when the cut spikes of gladiolus were treated with calcium exogenously^43^. Although a lower concentration of LG essential oil delayed floret opening but surprisingly reported a higher diameter. The increment in floral diameter is attributed to improved water uptake which keeps the petal tissues turgid and sustains the visual quality of cut flowers^16^.

RFW of individual gladiolus cut spike was significantly higher when treated with a lower concentration of LG essential oil than untreated control (Fig. 1D). Spikes treated with 5 µL L^−1^ LG essential oil reduced the transpiration rate and maintained the condition for uptake of water leading to prevent the RFW loss^44^. In addition, the longevity of cut flowers also depends upon the sugars level which serves as substrates for respiration, maintaining adequate water balance, providing structural support, and also acting as osmoregulatory substances in plants^45^. LG essential oils at lower concentrations maintained the adequate sugar concentrations in cut flowers resulting in increased RFW and delayed senescence. After day 9, when most of the open florets had undergone senescence, the carbohydrate content of the control flowers began to decline. As flower opening is known to be an energy-intensive process; sucrose decomposition and glucose consumption could reasonably account for the decrease. Moreover, the newly emerging flowers are the primary sinks for carbohydrates, while the senescent flowers are weaker in this regard^61^. Similarly, a significant effect of essential oil on RFW and the freshness of the flower was reported due to the increased sugar levels in the petals of lisianthus^7^.

As an important class of pigments carotenoids are pivotal in maintaining the integrity of membranes and take part in antioxidative defence in plants thus enhancing the ornamental quality of cut flowers^46^. In the present study, among all the treatments, the decrease in carotenoid content was less pronounced in flowers exposed to a lower doses of LG essential oil suggesting the slower rate of carotenoid degradation and hence a positive effect of LG essential oil in maintaining pigments of cut flowers (Fig. 5A). The application of essential oil has been proven beneficial in extending the vase life by maintaining pigments such as chlorophyll, and carotenoid in various cut flowers viz., chrysanthemum, alstroemeria, and gerbera^9,47,48^.

As a product of lipid peroxidation, MDA is a marker of oxidative stress induced damage to plant membranes^49^. Lesser accumulation of MDA content in cut spikes treated with LG essential oil (5 µL L^−1^) indicates a decrease in the level of lipid peroxidation thus maintaining the membrane stability and delaying the process of senescence. In addition, we have reported an increased SOD and CAT antioxidant enzyme activities in LG treated cut spikes. These enhanced antioxidant activities justified the role of antioxidants in cellular defence against oxidative stress and postharvest life^50^. These results are in accordance with the studies of other researchers who have reported the higher activity of antioxidative enzymes that scavenge the reactive oxygen species (ROS) to reduce the negative effects of oxidative stress in cut flowers such as gladiolus, sunflower, and carnation^51,52^.

Several previous investigations on molecular and regulatory networks involved in petal senescence have identified the involvement of various genes associated with senescence in flowers such as petunia, dianthus*,* and gladiolus. So far, there is no information about the molecular mechanisms of LG essential oil in the process of senescence in cut flowers. Thus, in the present study, we have investigated the effects of LG essential oil on the expression levels of genes involved in petal senescence (*GgCyP1*, *GgErs1a,* and *GgDAD*1) and antioxidant defence system (*GgP5C5, GgPOD 1, GgMnSOD, and GgCAT1)*. A variety of proteases and ubiquitin-mediated proteasomes are involved in senescence which leads to the degradation of proteins. An increase in *GgCYP1* and *GgErs1a* gene expression depicts the onset of senescence in its petals. In our findings, *GgCyP1* expression was highest in untreated control spikes, but the reduction in transcript levels was observed in florets of LG treated spike which delayed petal senescence (Fig. 6). Previous studies have also reported the role of cysteine protease in the senescence of cut flowers such as gladiolus, carnation, alstroemeria, and lilium^9,53–55^. Similar to the *GgCYP* gene, LG treatment has resulted in the reduction in expression of the *GgERS1a* in the cut flowers of ethylene-insensitive gladiolus as compared to control. Calcium treatment also led to the reduction in transcripts levels of senescence-associated genes viz., *GgCyP1* and *GgERS1a* at the time of flower development of gladiolus^56^. Current findings suggested that the expressions of the ethylene receptor promote senescence and its decline in expression delays senescence^57^. However, unlike *GgCYP1* and *GgErs1a*, *GgDAD1* (Defender of apoptotic death) expression is down-regulated in the petals in control on the fifth day and increased with the use of a low dose of LG essential oil in the vase solution. The results were in accordance with the studies conducted in Gladiolus where *DAD1* gene expression was highly reduced in fully wilted petals^58^. In comparison to other flowers crop species like iris and carnation, there is a downregulation of the expression of *DAD1* in petals during petal senescence.

The process of senescence involves progressive oxidative deterioration in the petals of cut flowers. As a defense mechanism, the antioxidant compounds increase during the onset of senescence but then decline gradually during advanced senescence^59^. This declination in the endogenous antioxidant level led to an increase in ROS and free radicals in the cut flowers. Therefore, there is a pressing need to identify potential antioxidant agents that can be supplemented exogenously in the vase solution to counteract the oxidative stress built during flower senescence. LG essential oil is an antimicrobial reagent that holds potential as an antioxidant and thus can be used as an elicitor of shelf-life elongation in cut flowers^60,61^. However, the expression pattern of antioxidant genes will be the same as the activity of their encoded enzymes. In the present study, the highest gene expression of different antioxidant enzymes *GgP5C5, GgPOD 1, GgMnSOD, and GgCAT1* could be detected in the 5 µL L^−1^ LG treated spikes as compared to control and other LG treated spikes, thus, we assumed that LG treatment delayed petal senescence by increasing gene expression of antioxidant enzymes. Previous studies reported the effect of various antimicrobial agents on stimulating the activity of antioxidant enzymes such as *POD1, MnSOD*, and *CAT1* thus increasing the vase life of cut flowers^62^.

## Conclusion

It is first to report that LG essential oil at 5 µL L^−1^ concentration can preserve the postharvest quality of cut spikes of gladiolus and extend their vase life. The positive effects of LG essential oil on vase life were attributed to maintained water relations, reduced microbial growth at the stem end, intact vasculature, maintained sugar level, carotenoids, decreased lipid peroxidation, enhanced antioxidant defense systems and reduced expression level of senescence-associated genes. Thus, this can be inferred that LG essential oil at an optimized concentration could be used as a novel eco-friendly flower preservative for commercial application in the cut gladiolus industry.

## Data Availability

The datasets generated during and/or analysed during the current study are publicly available from the corresponding author on request.
